# Effects of Bile Acid Modulation by Dietary Fat, Cholecystectomy, and Bile Acid Sequestrant on Energy, Glucose, and Lipid Metabolism and Gut Microbiota in Mice

**DOI:** 10.3390/ijms23115935

**Published:** 2022-05-25

**Authors:** Sunmin Park, Ting Zhang, Yu Yue, Xuangao Wu

**Affiliations:** 1 Department of Bio-Convergence System, Hoseo University, Asan 31499, Korea; zhangting92925@gmail.com (T.Z.); niyani0@naver.com (X.W.); 2 Obesity/Diabetes Research Center, Department of Food and Nutrition, Hoseo University, Asan 31499, Korea; yuyue6491@gmail.com

**Keywords:** high-fat diet, cholestyramine, gut microbiota, bile acid, dyslipidemia

## Abstract

Bile acid metabolism, involved with the digestion and absorption of nutrients in the gut, is linked to the gut microbiota community, greatly impacting the host’s metabolism. We examined the hypothesis that the modulation of bile acid metabolism by dietary fat contents, gallbladder removal (GBX; cholecystectomy), and bile acid sequestrant (BAS; cholestyramine) treatment could alter energy, glucose, and lipid metabolism through the changes in the gut microbiota. Mice were randomly assigned to the following six groups: (1) Sham GBX surgery (Sham) + low fat/high carbohydrate diet (LFD), (2) Sham + high fat diet (HFD), (3) Sham + HFD + BAS, (4) GBX + LFD, (5) GBX + HFD, and (6) GBX + HFD + BAS. BAS groups received 2% cholestyramine. After an 8-week intervention, energy, glucose, and lipid metabolism, and the gut microbiota community were measured. HFD groups exhibited higher body weight gain than LFD, and GBX increased the weight gain comped to Sham groups regardless of BAS in HFD (*p* < 0.05). Homeostatic model assessment for insulin resistance (HOMA-IR) was higher in HFD than LFD, and GBX increased it regardless of BAS. Serum lipid profiles were worsened in GBX + HFD compared to Sham + LFD, whereas BAS alleviated them, except for serum HDL cholesterol. Hepatic tumor-necrosis-factor-*α* (*TNF-α*) mRNA expression and lipid peroxide contents increased with GBX and BAS treatment compared to Sham and no BAS treatment (*p* < 0.05). Hepatic mRNA expression of sterol regulatory element-binding transcription factor 1c (*SREBP1c*) and peroxisome proliferator-activated receptor gamma (*PPAR-γ*) exhibited the same trend as that of tumor necrosis factor-α (*TNF-α*). The *α*-diversity of gut bacteria decreased in GBX + HFD and increased in GBX + HFD + BAS. *Akkermentia*, *Dehalobacterium*, *SMB53*, and *Megamonas* were high in the Sham + LFD, and *Veillonella* and *Streptococcus* were rich in the Sham + HFD, while *Oscillospira* and *Olsenella* were high in Sham + HFD + BAS (*p* < 0.05). GBX + LFD increased *Lactobacillus* and *Sutterella* while GBX + HFD + BAS elevated *Clostridium*, *Alistipes*, *Blautia*, *Eubacterium,* and *Coprobacillus* (*p* < 0.05). In conclusion, the modulation of bile acid metabolism influences energy, glucose, and lipid metabolisms, and it might be linked to changes in the gut microbiota by bile acid metabolism modulation.

## 1. Introduction

Gut microbiota plays a critical role in fermenting exogenous dietary fiber and endogenous mucus in the intestines. The fermentation supports the growth of microbes and the production of metabolites, including short-chain fatty acids (SCFA) [1]. Eliminating mucus and producing metabolites influences energy and glucose metabolism, inflammation, and immunity of the host, contributing to the host’s health [2]. Maintaining beneficial gut microbiota can prevent various metabolic diseases, including obesity, type 2 diabetes, non-alcohol hepatic steatosis, and Alzheimer’s disease [3,4]. Although the gut microbiome is not easily changed, environmental factors, such as diets, drugs, and physical activity, modulate the gut microbiota community [3,5]. Additionally, digestion and absorption processes, such as bile acid secretion, also influences gut microbiota composition [6].

Bile acid plays a crucial role in fat digestion and absorption in the gastrointestinal tract, and gallbladder diseases have been reported to modulate bile acid metabolism [7]. Bile acid is secreted into the intestinal lumen from the gallbladder, a reservoir of bile acid, by its contraction after fat consumption. It is reabsorbed in the distal ileum to return to the liver via the portal vein, known as enterohepatic bile acid circulation. In the liver, bile acids activate the Farnesoid-X-receptor (FXR) to inhibit cholesterol 7-hydroxylase, the rate-limiting enzyme of bile acid biosynthesis. FXR activation suppresses hepatic lipid biosynthesis by inhibiting sterol regulatory element-binding transcription factor 1c (*SREBP1c*) gene expression [7]. Furthermore, FXR activation inhibits gluconeogenesis and promotes glycogen and protein synthesis by activating glycogen synthase kinase-3 and ribosomal protein S6 and eukaryotic translation initiation factor 4B, respectively [8]. A small amount of primary bile acids enters the colon, changing into secondary and tertiary bile acids by gut bacteria. Therefore, bile acid suppresses hepatic lipid biosynthesis and glucose metabolism through FXR activation [9].

Treatment of bile acid sequestrant (BAS), such as cholestyramine, reduces dyslipidemia by suppressing the enterohepatic circulation of bile acids in the ileum. A higher percentage of bile acid goes to the large intestines to be changed into secondary bile acids. They are sent to the feces to remove them. BSA promotes bile acid synthesis from cholesterol in the liver and decreases serum LDL cholesterol concentrations [10]. However, long-term BSA treatment induces transient hypertriglyceridemia in healthy persons despite lowering serum cholesterol concentrations [10]. These effects are related to gut dysbiosis by increased bile acid. Bile acids in the gut are metabolized into secondary bile acids by gut microbiota, and it modulates diverse metabolic pathways to influence host metabolism [11]. Bile acids have antimicrobial activity by damaging the bacterial cell membrane, which suppresses bacterial overgrowth under normal conditions with small amounts of bile acids. In a high-fat diet, bile acid tolerable bacteria, such as *Bacteroides*, *Alistipes*, and *Bilophila* increase, whereas polysaccharide digesting bacteria, such as *Roseburia*, *Eubacterium rectale, Ruminococcus bromii,* and *Firmicute* decrease [12]. The overgrowth of increased bile acid tolerable bacteria triggers inflammatory bowel disease [12]. However, the link between diet and gut microbiota remains controversial: the effects of probiotics and prebiotics are not permanently colonized in gut microbiota [13].

The gallbladder concentrates and stores bile acid, released during fat intake to emulsify fats to be digested and absorbed. Gallbladder removal (GBX) prevents the regulation of bile acid release into the intestines during fat intake, and interestingly, it is associated with fatty liver, insulin resistance, metabolic syndrome, and obesity [9,14]. It suggests that the gallbladder has metabolic roles, and its removal might modulate the gut microbiota to modify the host metabolism. However, few studies have examined how bile acid metabolism in the gut influences gut microbiota, bidirectionally contributing to the host’s energy, glucose, and lipid metabolism. We examined the hypothesis that the modulation of bile acid content in the large intestines by dietary fat content, bile sequestrants (BAS), and cholecystectomy could alter energy, glucose and lipid metabolism, and gut microbiota in rodent animal models. Mice were used as an animal model in the present study since rats do not have a gallbladder [15].

## 2. Results

### 2.1. Energy Metabolism

A high-fat diet (HFD) increased body weight gain during 8-week experimental periods compared to a high carbohydrate/low fat (LFD). In contrast, GBX exhibited a higher weight gain than mice with intact gallbladders (Sham-operated mice) regardless of BAS treatment (Table 1). However, food intake was higher in LFD than in HFD regardless of GBX and BAS treatments, but daily caloric intake was similar in all groups (Table 1). Food efficiency was lower in LFD than in HFD, and GBX elevated it, but BAS did not alter food efficiency in HFD (Table 1). Visceral fat mass was elevated with HFD compared to LFD and was further increased in GBX but not BAS (Table 1). However, liver mass was higher in the LFD than HFD, and GBX, but not BAS, increased it (Table 1). Therefore, as expected, the HFD increased weight gain further by GBX as visceral fat mass, but those effects were not due to increased energy intake. 

### 2.2. Glucose and Lipid Metabolism

Serum glucose concentrations at a fasting state were not significantly different among all groups, but those in the post-prandial state were higher in GBX + HFD + BAS than in the other groups (Table 2). Homeostatic Model Assessment for Insulin Resistance (HOMA-IR; an insulin resistance index) was lower in the LFD than HFD, and GBX increased it regardless of BAS (Table 2). 

Serum total cholesterol concentrations were higher in the HFD than LFD, GBX decreased them, and BAS reduced them only in Sham rats but not GBX rats (Table 2). Serum HDL cholesterol concentrations were reduced in GBX rats compared to Sham rats regardless of BAS and dietary fat contents. Serum low-density lipoprotein (LDL) cholesterol concentrations showed a similar pattern to serum total cholesterol concentrations but were lowered with BAS in Sham and GBX rats (Table 2). However, serum triglyceride concentrations increased with GBX but decreased with BAS treatment. Serum triglyceride concentrations were higher in LFD than in HFD in Sham rats, but they were the opposite in GBX rats (Table 2). Therefore, GBX tended to raise cholesterol but lower triglycerides, whereas BAS lowered triglycerides. Since in GBX, bile acids were constantly released into the gut, this may inhibit cholesterol synthesis due to feedback from the enterohepatic circulation.

### 2.3. Hepatic Metabolism

Glycogen deposition in the liver was higher in LFD than HFD, GBX lowered it than Sham groups, and BAS did not alter it (Table 3). HFD did not influence triglyceride deposition compared to LFD. However, BAS increased triglyceride accumulation in the liver compared to no BAS treatment, and GBX increased triglyceride deposition only when combined with HFD without BAS (Table 3), suggesting that GBX could increase fat absorption. 

Tumor necrosis factor-α (*TNF-α*) mRNA expression was higher in GBX mice than in Sham mice, and it also increased in HFD compared to LFD (Table 3). BAS increased *TNF-α* expression only in GBX mice but not in intact mice. Hepatic *SREBP1c* mRNA expression was higher in the HFD + BAS than HFD, and GBX further elevated it. Hepatic SREBP1c mRNA expression was greatest in the GBX + HFD + BAS group. Hepatic triglyceride accumulation showed the same as hepatic *SREBP-1c* expression (Table 3). Peroxisome proliferator-activated receptor (*PPAR)-γ* mRNA expression was higher in the Sham + HFD than Sham + LFD, and GBX further elevated it. BAS treatment elevated its expression in the GBX group, but not the Sham group, compared to no BAS treatment (Table 3). The contents of lipid peroxide and TFN-α in the liver were higher in HFD than LFD in Sham and GBX mice, and BAS treatment did not alter their contents in Sham and GBX mice. Serum ALT concentration, an index of liver damage, was higher in GBX mice than in Sham mice, and BAS treatment further elevated it in GBX mice (Table 3). However, serum AST concentration was not altered with GBX and BSA treatment. Therefore, GBX, BAS, and their combination intervention increased the hepatic expression of fatty acid synthesis-related genes, *SREBP-1c* and *PPAR-γ*. These changes resulted in increased triglyceride accumulation, potentially elevating the mRNA expression of TNF-α, a pro-inflammatory cytokine. However, the decreased *Akkermansia* (a mucin degrading gut bacteria) may increase systemic inflammation and *TNF-α* expression. 

### 2.4. Histology of the Large Intestines

**Figure 1:**
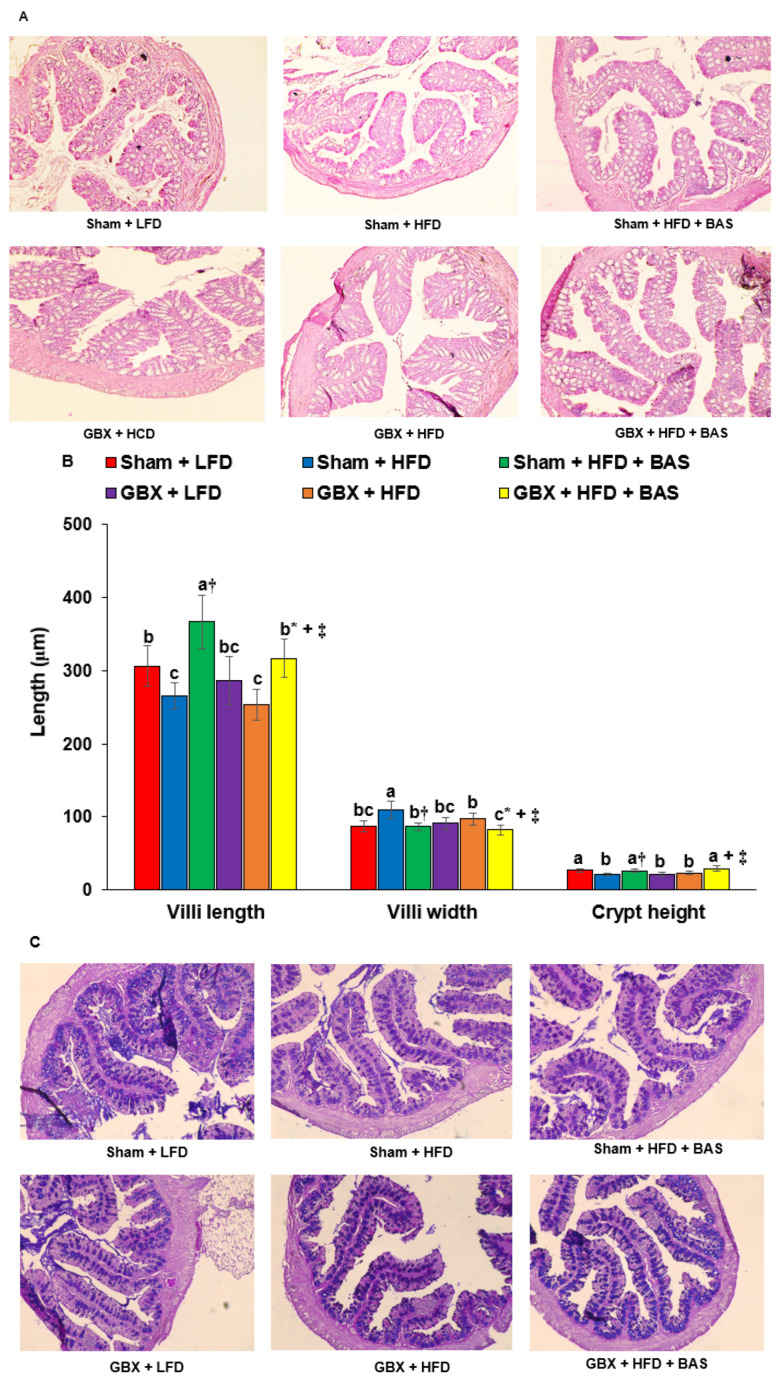

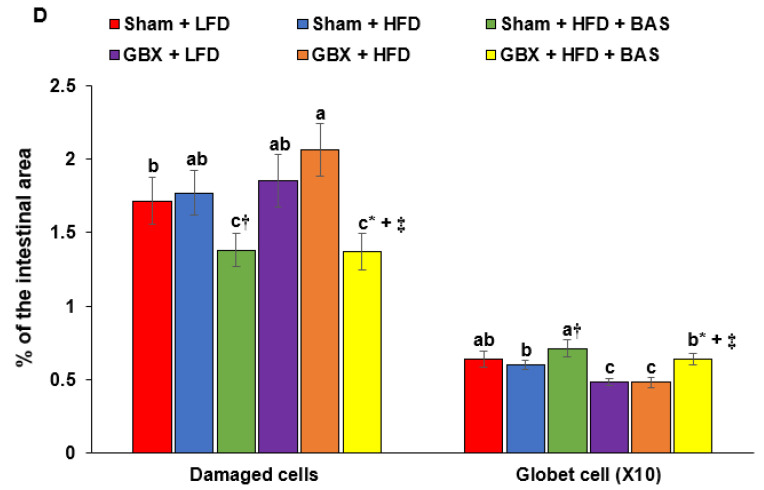
Areas of intestinal tissues and the amounts of goblet cells. (**A**) H-E stain picture. A red arrow indicated the villi. (**B**) The length and width of the intestinal villi. (**C**) Alcian blue-PAS staining picture. Blue spots were goblet cells with mucins, and the red arrow indicated the goblet cells. (**D**) The percentage of the damaged intestinal villi and impaired intestinal cells and the percentage of goblet cells. Mice were randomly assigned to the following six groups: (1) Sham operation of cholecystectomy (Sham) + low fat/high carbohydrate diets (LFD), (2) Sham + high fat diets (HFD), (3) Sham + HFD + 2% cholestyramine (BAS), (4) cholecystectomy (GBX) + LFD, (5) GBX + HFD, and (6) GBX + HFD + BAS. Feces were collected from the cecum after the 8-week treatment, and fecal bacterial composition was analyzed using the NGS method. Dots or bars and error bars represent the means ± standard deviations (*n* = 10). * Significant GBX effect in a two-way ANOVA at *p* < 0.05. ^+^ Significant treatment effect in a two-way ANOVA at *p* < 0.05. ^†^ Significant treatment effect in Sham rat by a one-way ANOVA at *p* < 0.05. ^‡^ Significant treatment effect in GBX rat by a one-way ANOVA at *p* < 0.05. a,b,c Different letters in each variable indicate significant differences in all groups at *p* < 0.05.

The intestinal villi length was shortened, and the empty space in the large intestines decreased in Sham + HFD compared to the Sham + LFD, and BAS treatment prevented the decrease. GBX deceased intestinal villi length in both LFD and HFD, whereas BAS treatment also prevented the decrement of the villi in the hematoxylin-eosin (H-E) staining (Figure 1A,B). In contrast to villi length, the villi width increased in Sham + HFD, GBX + HFD, and GBX + LFD, whereas GBX did not increase villi width despite shorter villi. BAS lowered villi width in Sham and GBX mice (Figure 1A,B). Crypt height was higher in Sham + HFD than Sham + LFD, and BAS prevented the decrease in crypt height. GBX reduced the crypt height in LFD and HFD, and BAS increased it as much as Sham + LFD (Figure 1A,B). 

The damage to cells in the large intestinal tissues did not differ between HFD and LFD, and BAS protected against the damage. GBX increased the intestinal cell damage, and BAS reduced it (Figure 1C,D). Furthermore, the number of mucin-producing goblet cells increased in Sham + HFD + BAS compared to Sham + HFD, while GBX reduced it compared to Sham in both LFD and HFD in Alcian blue-perchloric acid (AB-PAS) staining (Figure 1C,D). These results suggest that GBX, which results in a continuous flow of bile into the intestines, may cause damage to the gut cell wall either directly by damaging the cells or indirectly due to microbiota changes. BSA treatment can prevent the damage. 

### 2.5. Fecal Microbiota Community

**Figure 2:**
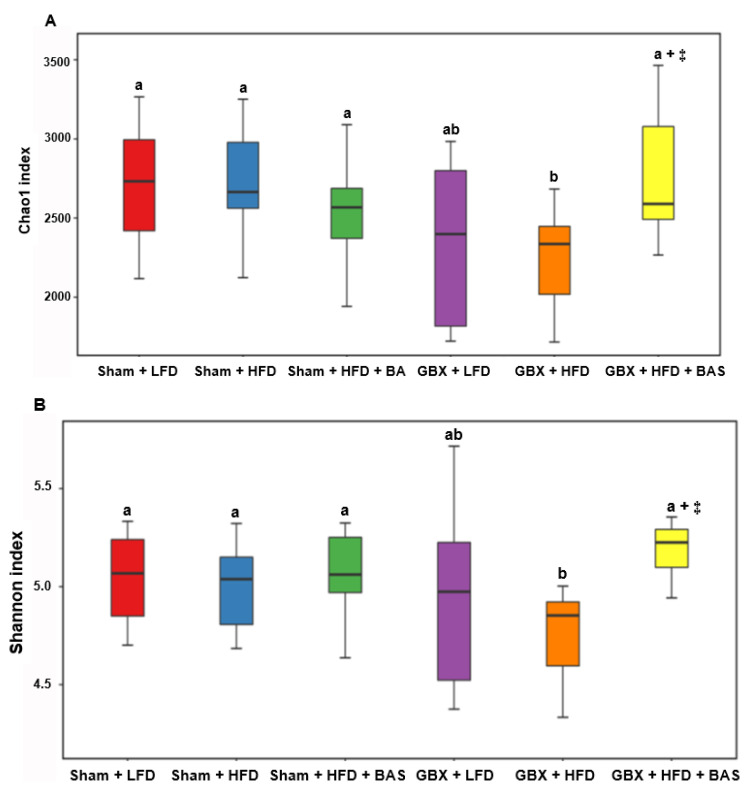

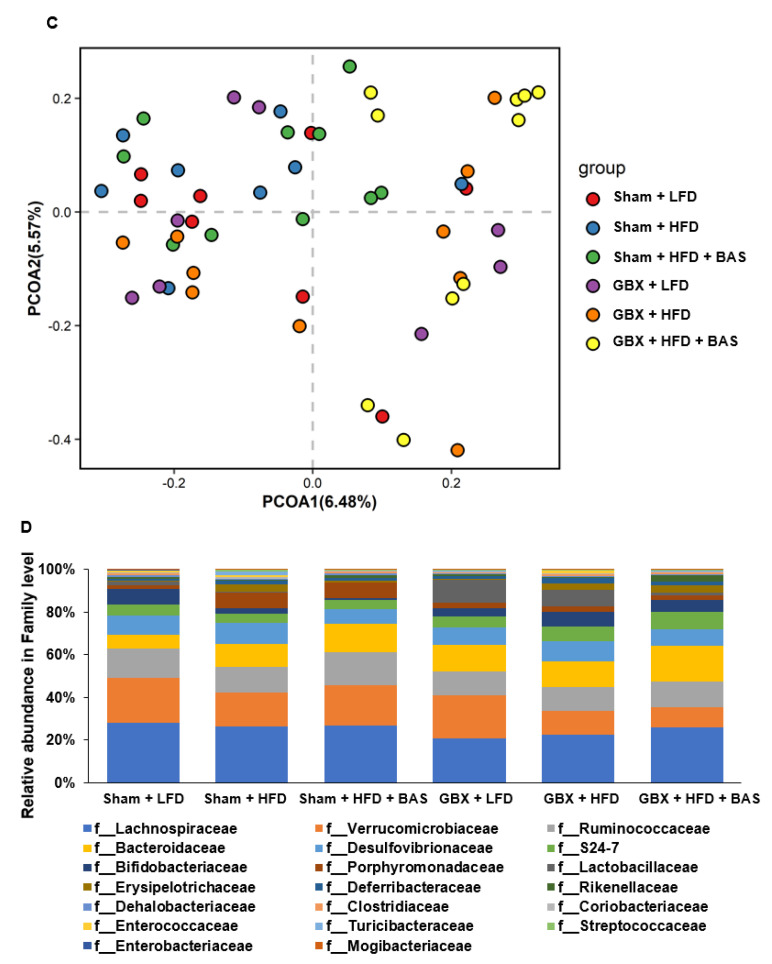

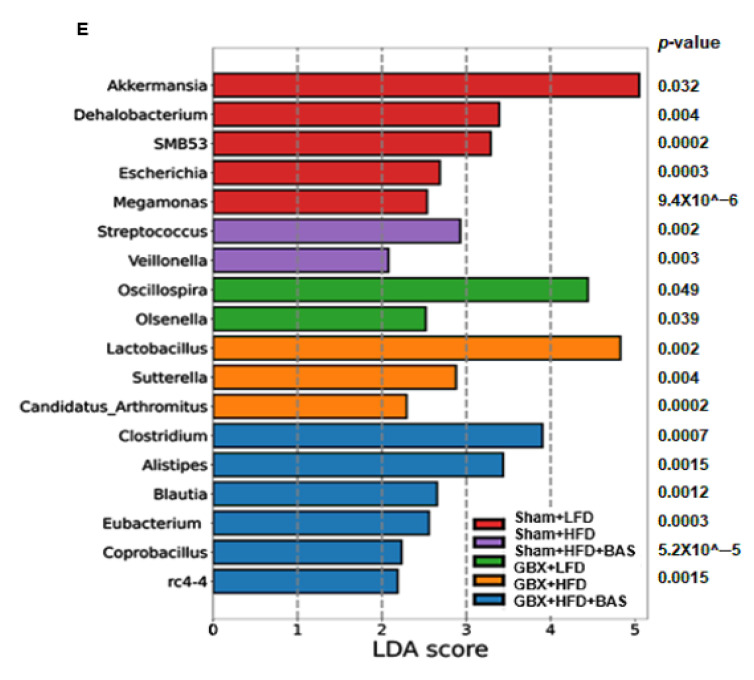
Gut microbiota α- and β-diversity and profiles. (**A**) α-diversity (Choa1 index) of gut microbiomes. (**B**) α-diversity (Shannon index) of gut microbiomes. (**C**) Fecal bacterial communities were separated by principal coordinate analysis (PCoA). (**D**) Relative abundance of gut microbiomes at the family level. (**E**) Linear discriminant analysis (LDA) scores of selected bacteria in each group. The bacteria represented in the LefSe were significantly different from the other groups at designated *p* values. Mice were randomly assigned to the following six groups: (1) Sham operation of cholecystectomy (Sham) + low fat/high carbohydrate diets (LFD), (2) Sham + high fat diets (HFD), (3) Sham + HFD + 2% cholestyramine (BAS), (4) cholecystectomy (GBX) + LFD, (5) GBX + HFD, and (6) GBX + HFD + BAS. Feces were collected from the cecum after the 8-week treatment, and fecal bacterial composition was analyzed using the NGS method. Dots or bars and error bars represent the means ± standard deviations (*n* = 10). ^+^ Significant treatment effect by a two-way ANOVA at *p* < 0.05. ^‡^ Significant treatment effect in GBX rat by a one-way ANOVA at *p* < 0.05. a,b Different letters in each variable indicate significant differences in all groups at *p* < 0.05.

The gut microbiota differences were linked to bile acid secretion and reabsorption. The α-diversity, according to Chao and Shannon indices, was lowest in GBX + HFD among all groups, but it was not significantly different in other groups (Figure 2A,B). These results suggested that in GBX + HFD, bile acid was insufficiently secreted and rapidly reabsorbed in the ileum. The decrement of bile acid decreased the α-diversity of gut microbiota in the large intestines. The β-diversity result showed that the Sham and GBX groups were significantly separated (Figure 2C). The microbiota data from the cecum revealed that Lachnospiraceae was lowered GBX + LFD and GBX + HFD than Sham + LFD and Sham + HFD. BAS treatment offset the decrease in Lachnospiraceae by GBX (Figure 2D). Verrucomicrobiaceae decreased in GBX with HFD diets regardless of BAS. Bacteroidaceae increased with HFD, GBX, and BAS treatments, and its abundance was highest in the GBX + HFD + BAS group among all groups (Figure 2D). At the genus level, *Akkermentia* was lowered more in GBX + HFD and GBX + HFD + BAS than others, while GBX and BAS treatment elevated *Bacteroides* (Table 4). *Lactobacillus* was higher in GBX + LFD and GBX + HFD than in Sham + HFD and Sham + HFD, but BAS treatment in GBX did not increase *Lactobacillus* (Table 4). 

LDA scores also showed the bacteria with linear discriminant analysis (LDA) scores > 2 at the genus level, and GBX + HFD did not have bacteria meeting the criteria of LDA scores (Figure 2E). *Vellonella* and *Streptococcus* were rich in the Sham + HFD, while *Akkermentia*, *Dehalobacterium*, *SMB53*, and *Megamonas* were high in the Sham + LFD. *Oscillospira* and *Olsenella* were high in Sham + HFD + BAS (Figure 2E). In GBX mice, LFD increased *Lactobacillus* and *Sutterella* while HFD + BAS elevated *Clostridium, Alistipes, Blautia, Eubacterium*, and *Coprobacillus* (Figure 2E). 

### 2.6. SCFA and Metagenome Functions of Fecal Bacteria

**Figure 3:**
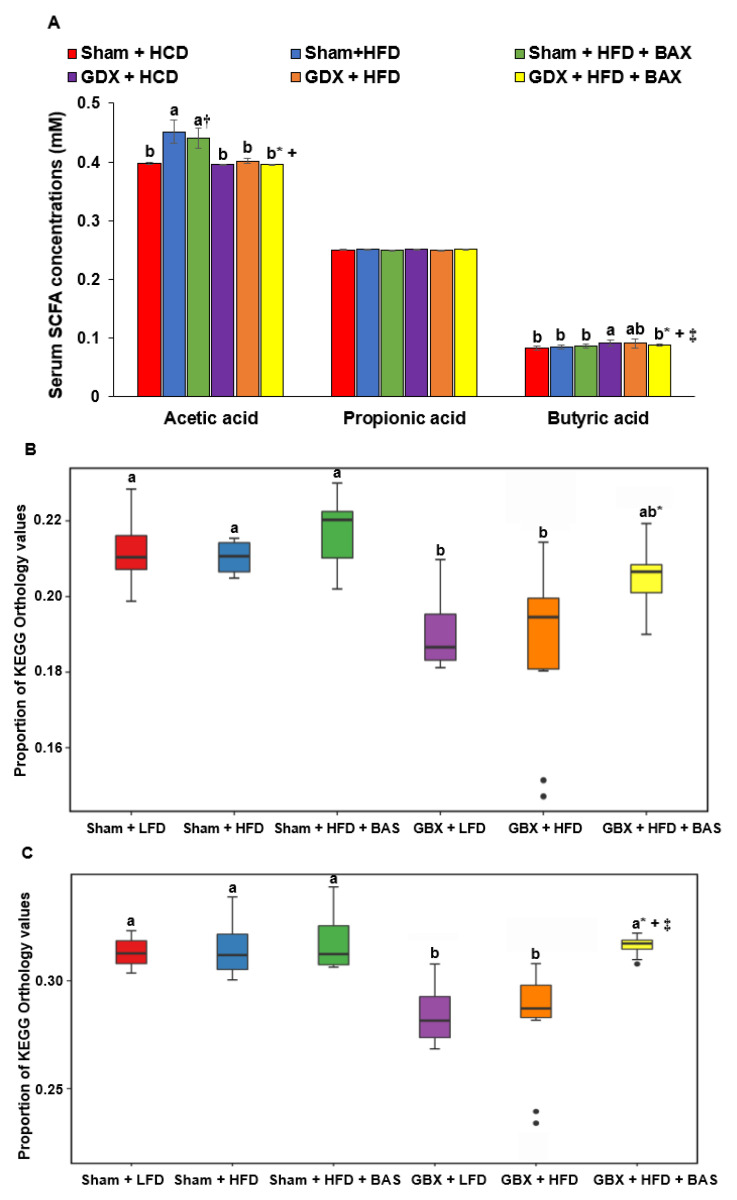

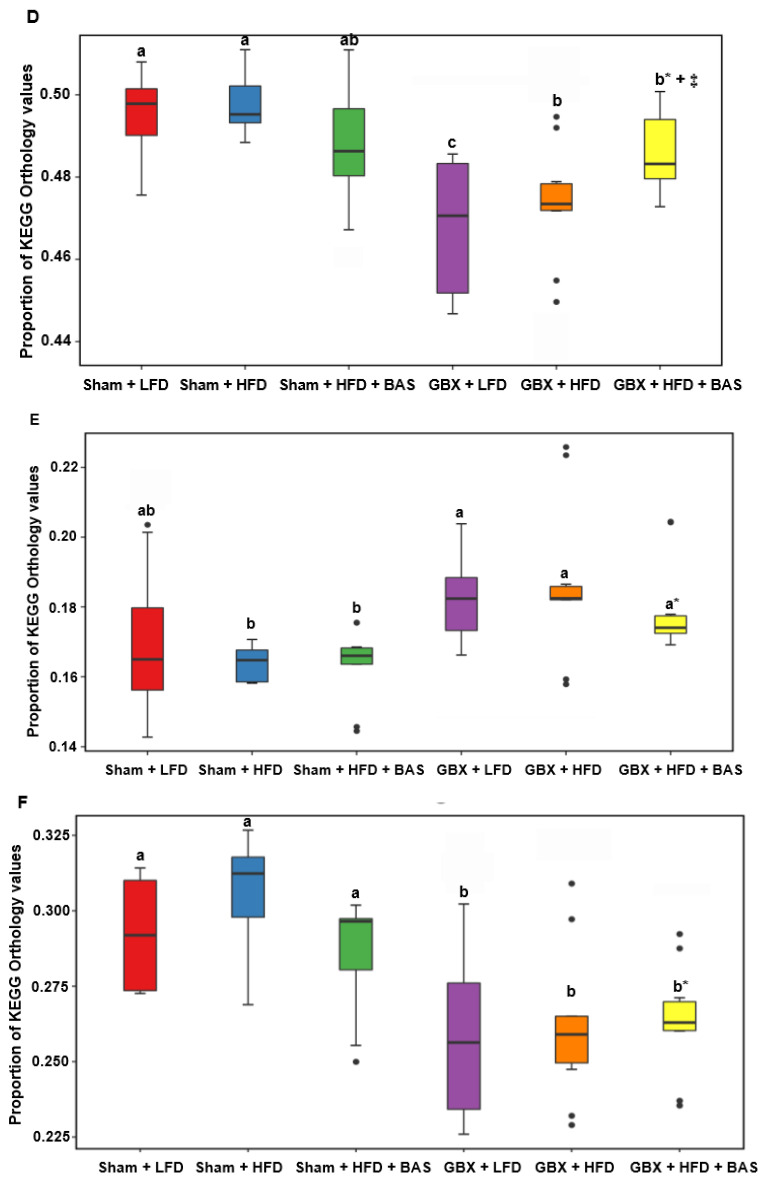
Short-chain fatty acids and prediction of gene function in the fecal bacteria by PICRUSt2. (**A**) Short-chain fatty acid concentrations in the portal vein measured by gas chromatography. (**B**) Proportion of KEGG Orthology (KO) values of citrate cycle, first oxidation, oxaloacetate → 2-oxoglutarate. (**C**) The proportion of KEGG Orthology (KO) values of leucine biosynthesis, 2-oxoisovalerate → 2-oxoisocaproate. (**D**) The proportion of KEGG Orthology (KO) values of isoleucine biosynthesis, threonine → 2-oxobutanoate → isoleucine. (**E**) The proportion of KEGG Orthology (KO) values of cysteine biosynthesis, methionine → cysteine. (**F**) The proportion of KEGG Orthology (KO) values of methionine degradation. Mice were randomly assigned to the following six groups: (1) Sham operation of cholecystectomy (Sham) + high carbohydrate diets (LFD), (2) Sham + high fat diets (HFD), (3) Sham + HFD + 2% cholestyramine (BAS), (4) cholecystectomy (GBX) + LFD, (5) GBX + HFD, and (6) GBX + HFD + BAS. Feces were collected from the cecum after the 8-week treatment, and fecal bacterial composition was analyzed using the next-generation sequencing method. Each bar and error bar represented the means ± standard deviations (*n* = 10). * Significant GBX effect by a two-way ANOVA at *p* < 0.05. ^+^ Significant treatment effect by a two-way ANOVA at *p* < 0.05. ^†^ Significant treatment effect in Sham rat by a one-way ANOVA at *p* < 0.05. ^‡^ Significant treatment effect in GBX rat by a one-way ANOVA at *p* < 0.05. a,b,c Different letters in each variable indicate significant differences in all groups at *p* < 0.05.

SCFA concentration in the portal vein primarily originated from gut microbiota. Acetate concentration in the portal vein was higher in the Sham + HFD and Sham + HFD + BAS than in other groups, whereas the butyrate concentration was higher in GBX + LFD (Figure 3A). However, propionate concentration did not differ among the groups (Figure 3A). These results suggested that high bile acid content in the cecum increased acetic acid concentration production by microbiota. When there is a high bile acid, bacteria growth, especially butyrate and propionate-producing bacteria, may be inhibited, and in the opposite condition, bacteria may overgrow, especially harmful bacteria. Bile acid secretion and enterohepatic circulation regulate bile acid content, and optimal contents of bile acid promote gut microbiota, contributing to improving the host’s metabolism. 

The metagenome function of fecal bacteria as determined with the Picrust2 program is presented in Figure 3B–E. The citrate cycle related to energy metabolism was lower in the GBX groups than in the Sham groups (Figure 3B), suggesting that higher body weight gain in the GBX groups might be associated with different gut microbiota between Sham and GBX groups. However, LFD and HFD did not differ in the citrate cycle of the fecal bacteria, and BAS did not show a significant difference (Figure 3B). Leucine and isoleucine biosynthesis, branched-chain amino acids, was higher in the Sham groups than GBX + LFD and GBX + HFD but not GBX + HFD + BAS (Figure 3C,D). However, cysteine biosynthesis was lower in the Sham groups than in the GBX groups (Figure 3E), while methionine degradation was opposite to cysteine biosynthesis (Figure 3F).

**Figure 4:**
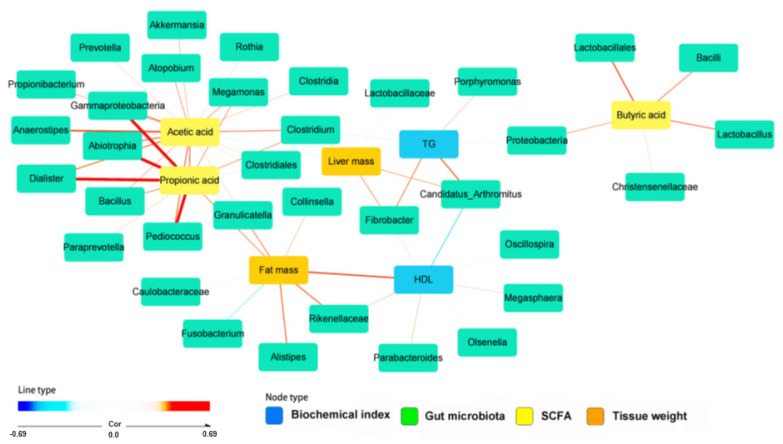
Network analysis of fat metabolism-related parameters, SCFA, and gut bacteria. The thin lines indicated the correlation of two corresponding parameters at *p* < 0.05 and the thick lines at *p* < 0.001. Red and blue lines indicated positive and blue associations, respectively. SCFA, short-chain fatty acids. Cor, correlation coefficient.

Network analysis of fat metabolism-related parameters, SCFA, and gut bacteria are given in Figure 4. Butyric acid was positively associated with *Bacillus, Lactobacillus*, Christensenellacae, and proteobacteria. Acetic acid was partly overlapped with propionic acids in Clostridium, SMB53, Propionibacterium, and Gammaproteobacteria. Acetic acid was strongly and positively associated with *Prevotella, Rothia, Clostidales*, and *Collinsella* and it was also positively associated with *Akkermentia, Bacillus, Pedioccocus*, and *Clostridium*. Acetic and propionic acids were positively associated with fat mass but negatively correlated with liver mass (Figure 4). Serum triglyceride concentration was positively correlated with Clostridium, Proteobacteria, and Porphyromonas. Serum HDL cholesterol concentrations were strongly linked to *Olsenella* and *Oscillospira,* and they were related to *Parabacteriodes, BF311*, and *Megasphaera*. The network analysis suggested that many gut microbiota strongly influence acetic acid, which was linked to acetic acid, and acetic acid is positively associated with fat mass and negatively linked with liver mass. Therefore, the gut microbiota is strongly associated with SCFA and lipid metabolism.

## 3. Discussion

Gut microbiota influences the host’s metabolism, and eubiosis plays a crucial role in maintaining good health. Although gut microbiota is affected by long-term dietary patterns and lifestyles, it is also inherited. In addition to dietary composition, the gut microbiota can be affected by digestive juice secretion and its reabsorption, which can change the pH in the gut. HFD is reported to cause a rapid increase in the intestinal bile acid pool within 12 h and an alteration in microbial composition at 24 h [16]. Bile acid metabolism greatly influences gut microbiota and intestinal bile acid modulation by dietary fat. Therefore, HFD and GBX might change gut microbiota to disturb energy, glucose, and lipid metabolism regulation. In the present study, fat intake, GBX, and BAS treatment changed bile acid contents in the cecum: GBX and HFD disturbed energy, glucose, and lipid metabolism, and BAS intervention in Sham mice, but not GBX mice, alleviated the disturbance. It is also related to gut microbiota changes potentially due to modulation of bile acid metabolism. GBX + HFD, but not GBX + LFD, lowered the α-diversity, and BAS treatment in GBX + HFD protected against it. HFD increased weight gain, insulin resistance, and lipid profiles compared to LFD, but the α-diversity of gut microbiota did not differ between HFD and LFD. 

Bile acid secretion and enterohepatic circulation regulate lipid metabolism [17]. Bile acid acts as an emulsifier of fat to be absorbed and a regulator of energy, glucose, and lipid metabolism. Bile acid modulates FXR and Takeda G-protein-coupled receptor 5 signaling to regulate gut hormone secretion (such as glucagon-like peptide-1 and peptide YY hepatic gluconeogenesis), glycogen synthesis, energy expenditure, and gut microbiota composition [18]. HFD secretes cholecystokinin (CCK) to release bile acid to contract the gallbladder. CCK acts as a regulator of energy and glucose metabolism by delivering the metabolic message from the liver to the brain [19]. Furthermore, bile acid can be a signal molecule of the brain-liver-gut axis. Its disturbance results in metabolic syndromes, such as obesity, fatty liver, and type 2 diabetes [20]. Consistent with the present study, HFD increases hepatic gluconeogenesis and fat synthesis, elevating insulin resistance, compared to LFD [20]. HFD increased Bacteroides but decreased *Akkermentia*, *Bifidobacterium*, and *Lactobacillus* to less than LFD. The differences were primarily consistent with Wang et al. [21], who showed similar differences in abundance in mice to the present study. However, they showed higher α-diversity in HFD, unlike our results. However, the results remain inconsistent with some other studies [22,23]. HFD slightly increases the total bile acid pool in the liver and feces, and their deoxycholic acid and taurodeoxycholic acid contents are higher in HFD than in LFD [24]. Therefore, the bile acid metabolism capacity of the host in each diet may play a key role in determining the gut microbiota community. 

The gallbladder acts as a storage and concentration site for bile acids produced from the liver. It releases bile acid by contraction and then refills in response to signals from the gut hormones cholecystokinin and fibroblast growth factors 15/19, respectively, when exogenous fat enters the intestines [17]. GBX is reported to increase the risk of metabolic syndrome, including abdominal obesity, hyperglycemia, dyslipidemia, hypertension, and non-alcoholic fatty liver [14]. The present confirmed that it might impair energy, lipid, and glucose metabolisms to induce metabolic syndrome and non-alcoholic fatty liver in mice. The disturbance of metabolism by GBX may be related to the changes in gut microbiota. GBX is known to disrupt enterohepatic recycling of and signaling by bile acid, contributing to the development of metabolic syndrome [25]. GBX may change bile acid contents in the gut to modulate gut microbiota. However, bile acid composition and amount in the gut remain controversial. 

GBX decreases the host’s bile acid pool size and disturbs the enterohepatic circulation of bile acids; as a result, it increases the exposure of intestinal bacteria to bile acids [25]. GBX increases the proportion of secondary bile acids by bacterial deconjugation and dihydroxylation [26]. However, some studies have reported that GBX does not change bile acid contents in the intestines since it may suppress the enterohepatic circulation [27]. The present study showed that GBX changed gut microbiota, decreased *Akkermentia,* and increased *Lactobacillus*. GBX + HFD + BAS increased *Balutia* and *Allstipes.* A previous study also showed that GBX increases *Blautia obeum* and *Veillonella parvula* were more abundant in the cholecystectomy group [26]. GBX with HFD exacerbated gut microbiota compared to GBX with LFD. Persons with GBX are advised to consume a low-fat diet to improve metabolism by promoting eubiosis. These results indicated that bile acids released into the cecum might modulate the microbiota community. 

BAS mainly lowers LDL cholesterol between 15% and 30% and slightly increases serum HDL concentrations by 3–5% [28]. However, their effect on serum triglyceride concentrations remains controversial [10]. The present study showed BAS lowered serum total and LDL concentrations in HFD-fed mice with intact gallbladders but not in GBX mice. Serum HDL concentrations tended to increase in Sham + HFD + BAS compared to Sham + HFD. However, BAS did not alleviate dyslipidemia in GBX mice. Sham + HFD + BAS increased *Bacteroides* and reduced *Desulfovibrio* compared to Sham + HFD. BAS treatment results in the excretion of bile acids in feces, and it changes compositional and functional changes of bile acids and gut microbiota in the gut [29]. In GBX, BAS treatment increased α-diversity compared to no BAS treatment in HFD, but it reduced beneficial *Lactobacilli* and increased pathogenic *Eubacterium* and *Clostridium*. Therefore, proper amounts of bile acid contents in the cecum might improve the gut microbiota. 

This study had some potential limitations. The microbial analysis was based on 16S rRNA sequencing, and their function was evaluated by Picrust2 analysis. Short-gun sequencing analysis may provide a more accurate microbial function. Furthermore, we compared the LFD, HFD, and HFD + BAS groups as individual treatment groups in Sham and GBX mice, and some comparison groups, such as LFD + BSA in Sham and GBX mice, were not included due to low secretion of bile acid in LFD. Therefore, further experiments are needed to confirm the results. Finally, the ability to translate rodent data to humans is questionable, and the study needs confirmation in human subjects. 

In summary, bile acid functions to facilitate the absorption of lipids, and it also acts as a regulator of energy, glucose, and lipid metabolism. The modulation of bile acid metabolism altered its availability in the large intestines, linked to gut microbiota changes. GBX impaired energy, glucose, and lipid metabolism, and BAS in GBX mice did not improve the metabolism. BAS increased the expression of *TNF-α*, the pro-inflammatory cytokine in GBX mice, which might be linked to its increased expression of genes related to fatty acid synthesis and/or decreased *Akkermansia* in the gut. BAS in GBX mice might dysregulate bile acid metabolism to impair hepatic lipid metabolism and gut microbiota disturbance. The metabolic effects of bile acid modulation may be linked to changes in the gut microbiota induced by increased bile acid availability in GBX and BAS intervention. In conclusion, bile acid modulation by HFD, GBX, and BAS alters energy, glucose, and lipid metabolism, and it was closely linked to promoting dysbiosis of the gut microbiota community. 

## 4. Materials and Methods

### 4.1. Animal Care

Sixty male ICR mice aged seven weeks (35.2 ± 0.98 g) were purchased from DaehanBio (Eumsung, Korea) and acclimated to the animal facility for one week. They were raised at 23 °C, 60% humidity, and a 12-h light/dark cycle, freely consuming food and water. The study was conducted according to the National Institute of Health Guidelines and was approved by the Hoseo University Animal Care and Use Committee (HSIAUC-17023). 

### 4.2. Diet Preparation

A high-fat diet (43 energy percent (En%) fat) and a low-fat diet (20 En% fat) were made with semi-purified methods. A high-fat diet contained 37 En% carbohydrates (cornstarch and sucrose), 20 En% protein (casein), and 43 En% lard (CJ Co., Seoul, Korea); a low-fat diet included 60 En% carbohydrates (cornstarch and sucrose), 20 En% protein (casein), and 20 En% lard (CJ Co., Seoul, Korea). Cellulose (2.5%) as dietary fiber and cholesterol (2.5%) were identical in all diets. The recommended amounts of vitamins and minerals were added. Cholestyramine (bile acid sequestrant; 2% *w*/*w*) was supplemented in a high-fat diet in the BAS group, and starch instead of cholestyramine was added in the other groups. Caloric density was higher in HFD (5.08 kcal/g) than in LFD (4.38 kcal/g). Feeds were then re-sieved and stored at 4 °C. All rats had free access to water and experimental diets during the entire experimental period. 

### 4.3. GBX (Cholecystectomy)

Overnight-fasted animals were anesthetized with ketamine and xylazine (100 and 10 mg/kg body weight), and the abdominal skin was dissected. The cystic duct was ligated, and the gallbladder was emptied with a 24-gauge needle syringe. The gallbladder was gently removed by surgical dissection, and the abdominal skin was closed by suture with silk. Sham groups had a sham cholecystectomy operation by gentle scrubbing the gallbladder without the removal. 

### 4.4. Experimental Design

**Figure 5:**
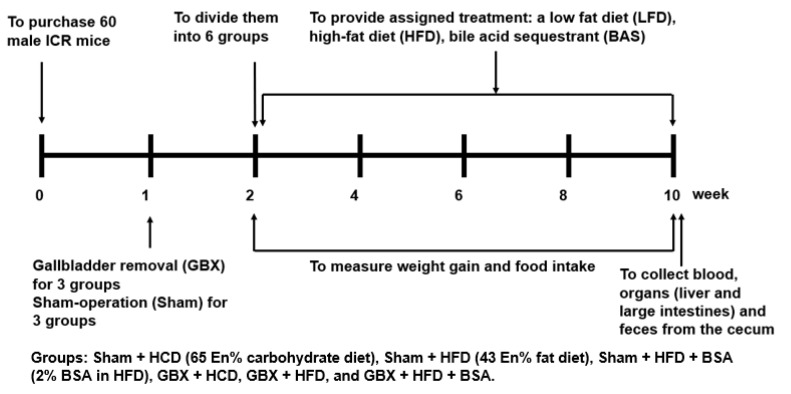
Experimental scheme.

The experimental design is presented in Figure 5. After 1-week recovery from the surgery, sham-operated and GBX mice were randomly assigned to the following six groups: (1) sham cholecystectomy operation (Sham) + LFD (Sham + LFD), (2) Sham + HFD, (3) Sham + HFD + BAS, (4) GBX + LFD, (5) GBX + LFD, (6) GBX + HFD + BAS. They had free access to their assigned diets for eight weeks, and at the end of the experiments, they had a 16 h fasting. After anesthetizing the mice with ketamine and xylazine, blood samples were drawn from the portal vein and the inferior vena cava. The organs were collected and rapidly frozen with liquid nitrogen. The feces in the cecum were collected and frozen at −70 °C. 

Liver glycogen contents were measured after lysis of the liver, and the supernatants were deproteinized with 1.5 N perchloric acid. The glycogen content was calculated from glucose concentrations derived from hydrolyzing glycogen by α-amyloglucosidase in an acid buffer [30]. Glucose concentrations were measured with a glucose kit (Asan Pharmaceutics, Seoul, Korea). Triglyceride storage in the liver was extracted with chloroform–methanol (2:1, *v*/*v*) from the hippocampus and resuspended in pure chloroform [31], and triglyceride in the chloroform was assessed by Triglyceride colorimetric kit (Asan Pharmaceutics, Seoul, Korea). Lipid peroxide contents in the liver were measured by measuring thiobarbituric acid reactive substances (TBARS) using TBARS assay kits (Abcam, Cambridge, UK) [32]. Serum TNF-α concentrations were measured using an ELISA kit (eBioscience; San Diego, CA, USA). Serum AST and ALT concentrations were measured using Colorimetry AST and ALT kits (Asan Pharmaceutics, Seoul, Korea). The assays using the kits were conducted according to the manufacturer’s manual.

### 4.5. Quantitative Real-Time PCR

Total RNA was extracted from the liver with Trizol reagent (Invitrogen, Rockville, MD, USA). cDNA was synthesized with an isolated total RNA, superscript III reverse transcriptase, and high fidelity Taq DNA polymerase (1:1:1, *v*/*v*/*v*) using the polymerase chain reaction (PCR) method. The cDNA was mixed with the primers for the genes of interest and SYBR Green mix to determine the expressions of the designated genes using a real-time PCR machine (BioRad Laboratories, Hercules, CA, USA). The primers used for *SREBP1c*, *PPAR-γ*, *TNF-α*, and *β-actin* are provided in previous study [33]. The gene expression levels were quantitated using the comparative cycle of threshold method [34]. 

### 4.6. Histology of the Large Intestines

After anesthetizing the mice, one lobe of the liver was dissected, and they were sequentially perfused with saline and a 4% paraformaldehyde solution (pH 7.2). The large intestinal tissues were immediately dissected and post-fixed with 4% paraformaldehyde overnight at room temperature [35]. Two serial 5-μm paraffin-embedded large intestine sections were randomly chosen, and they were subjected to H-E and AB-PAS staining. After staining, the area of intestinal villi was measured in H-E stained sections using a Zeiss Axiovert microscope with DIXI Imaging solution at 10× magnification. The impaired cells were counted in the H-E section, and the relative area and the number of impaired cells were scored 0–5. The intestinal goblet cells producing mucin, indicated by blue staining, were counted using the AB-PAS stained sections. 

### 4.7. Serum SCFA Concentrations and Gut Microbiome by Next-Generation Sequencing (NGS)

Serum separated from the portal vein blood was mixed with ethanol (Duksan, Korea), and 1 N HCl (100:1) was added to the mixture. It was vortexed and centrifuged at 15,000 rpm, 15 min, and 4 °C. SCFA concentrations in the supernatants were measured by gas chromatography (GC, Clarus 680 GAS, PerkinElmer, Boston., MA, USA) containing an Elite-FFAP 30 m × 0.25 mm × 0.25 μm capillary column. Helium was used as the carrier gas at a flow rate of 1 mL/min, as described previously [36]. Exogenous acetate, propionate, and butyrate (Sigma Co., St. Louis, MO, USA) were used as external standards.

Fecal microbiome communities were measured in the cecum using next-generation sequencing procedures [37]. According to the manufacturer’s instructions, bacterial DNA was extracted from feces using a Power Water DNA Isolation Kit (Qiagen, Valencia, CA, USA). DNA was amplified with 16S amplicon primers by PCR, and libraries were prepared for PCR products according to the GS FLX plus library prep guide, as described previously [38]. According to the manufacturer’s instructions, the PCR amplification program was run with 16S universal primers in the FastStart High Fidelity PCR System (Roche, Basel, Switzerland). Sequencing of bacterial DNA in feces was conducted using the Illumina MiSeq standard operating procedure and a Genome Sequencer FLX plus (454 Life Sciences) (Macrogen Inc., Seoul, Korea).

16S amplicon sequences were processed using Mothur v.1.36. Miseq SOP was used to identify fecal bacterial taxonomy, and bacterial counts were conducted on each fecal sample collected from the cecum. Sequences were aligned using Silva reference alignment v.12350. In a preclustering step, the sequences with an identity of ≥99% were merged. The chimeric sequences were detected and discarded by UCHIME. All the sequences were assigned to taxonomic classifications using Greengenes 13_8_99, and the sequences classified as mitochondria, Eukaryota, or unknown were removed. We conducted the picking of operational taxonomic units (OTUs) delimited at 98% identity, which was taxonomically classified by consensus using Greengenes 13_8_99. A relaxed neighbor-joining tree with one representative sequence per OTU was obtained with Clearcut after calculating uncorrected pairwise distances between aligned reads [36,38]. The relative number of bacteria was calculated in the taxonomic assignments at the family, order, and genus levels after removing operational taxonomic units (OTUs) below 10,000 reads. Principle component analysis (PCA) was conducted using the R package with the OTU-abundance table converted to relative abundance. The α-diversity Chao and Shannon indices were calculated using the Mothur summary.single subroutine. PCoA results for gut bacteria were visualized using the R package. 

### 4.8. Determination of Metabolic Functions of the Gut Microbiome by PICRUSt2 Pipeline Analysis

Metabolic functions of gut microbiota were predicted from the fasta files and count tables of fecal bacteria using PICRUSt2 [39]. Metabolic functions were predicted using the Kyoto Encyclopedia of Genes and Genomes (KEGG) Orthologues (KO) mapped by the KEGG mapper (https://www.genome.jp/kegg/tool/map_pathway1.html, accessed on 22 November 2021) [38]. The gut microbiome was used to explore the differences in metabolic functions among the groups.

Network analysis determined the links among gut bacteria at the genus level, SCFA, metagenome functions, and lipid metabolism-related parameters were determined [40]. 

### 4.9. Statistical Analyses

The statistical analysis was performed using SAS Version 7 (SAS Institute; Cary, NC, USA). A sample size of 10 per group was determined using the G power program (power = 0.85 and effect size = 0.50) to test the main effects. Results are expressed as means ± standard deviations (SDs). Univariate analysis was used to analyze normally distributed variables. A two-way analysis of variance (ANOVA) was conducted to compare GBX and treatment (dietary fat content and BSA) effects. The significant treatment effect was conducted with one-way ANOVA separately in each GBX and Sham group. Multiple comparisons were conducted with the Tukey test when ANOVA showed a significant intergroup difference. Statistical significance was accepted for *p* values < 0.05.

## Figures and Tables

**Table 1:** Body weight, food efficiency, and visceral fat mass.

	Sham + LFD (*n* = 10)	Sham + HFD (*n* = 10)	Sham + HFD + BAS (*n* = 10)	GBX + LFD (*n* = 10)	GBX + HFD (*n* = 10)	GBX + HFD + BAS (*n* = 10)
Final body weight (g)	36.0 ± 1.86 ^b^	37.6 ± 2.8 ^ab^	39.0 ± 2.8 ^a†^	36.4 ± 1.52 ^b^	39.6 ± 3.48 ^a^	39.8 ± 3.64 ^a^*^+^^‡^
Body weight gain (g)	7.1 ± 0.61 ^c^	9.0 ± 1.1 ^b^	9.1 ± 0.44 ^b†^	8.4 ± 0.60 ^b^	11.8 ± 0.55 ^a^	12.4 ± 0.80 ^a*+^^‡^
Food intake (g/day)	6.09 ± 0.53 ^a^	4.59 ± 0.59 ^b^	4.71 ± 0.51 ^b†^	5.75 ± 0.51 ^a^	4.89 ± 0.56 ^b^	5.03 ± 0.66 ^b*^^‡^
Caloric intake (kcal/day)	26.7 ± 2.76	23.3 ± 3.00	23.9 ± 2.59	25.2 ± 2.50	24.8 ± 2.84	25.6 ± 3.35
Food efficiency (g/day)	1.17 ± 0.44 ^c^	1.84 ± 0.54 ^b^	1.85 ± 0.58 ^b†^	1.33 ± 0.51 ^c^	1.98 ± 0.69 ^ab^	2.49 ± 0.64 ^a^*^+^^‡^
Visceral fat mass (g)	1.42 ± 0.18 ^c^	1.74 ± 0.20 ^b^	2.26 ± 0.22 ^a†^	1.67 ± 0.21 ^b^	2.43 ± 0.58 ^a^	2.31 ± 0.30 ^a^*^+^^‡^
Liver mass (g)	1.89 ± 0.07 ^b^	1.63 ± 0.06 ^c^	1.66 ± 0.07 ^c†^	2.06 ± 0.17 ^a^	1.91 ± 0.20 ^ab^	1.91 ± 0.14 ^ab^*^+^

Mice were randomly assigned to the following six groups: (1) Sham operation of cholecystectomy (Sham) + low fat/high carbohydrate (LFD), (2) Sham + high fat diets (HFD), (3) Sham + HFD + 2% cholestyramine (BAS), (4) cholecystectomy (GBX) + LFD, (5) GBX + HFD, and (6) GBX + HFD + BAS. Values represented means ± standard deviations (*n* = 10). * Significant GBX effect by a two-way ANOVA at *p* < 0.05. ^+^ Significant treatment effect by a two-way ANOVA at *p* < 0.05. ^†^ Significant treatment effect in Sham rat by a one-way ANOVA at *p* < 0.05. ^‡^ Significant treatment effect in GBX rat by a one-way ANOVA at *p* < 0.05. ^a,b,c^ Different letters in each variable indicate significant differences in all groups at *p* < 0.05.

**Table 2:** Glucose and lipid metabolism in the circulation.

	Sham + LFD (*n* = 10)	Sham + HFD (*n* = 10)	Sham + HFD + BAS (*n* = 10)	GBX + LFD (*n* = 10)	GBX + HFD (*n* = 10)	GBX + HFD + BAS (*n* = 10)
Fasting serum glucose (mg/dL)	93.0 ± 8.8	85.9 ± 8.54	86.3 ± 6.32	82.3 ± 8.78	87.9 ± 6.83	92.3 ± 8.93
Post-prandial serum glucose (mg/dL)	168 ± 13.4 ^b^	157 ± 10.6 ^b^	161 ± 7.89 ^b^	160 ± 9.1 ^b^	168 ± 11.1 ^b^	182 ± 10.9 ^a^*^+^^‡^
Fasting serum insulin (ng/mL)	1.05 ± 0.21 ^d^	1.54 ± 0.27 ^b^	1.51 ± 0.24 ^b†^	1.33 ± 0.23 ^c^	1.81 ± 0.27 ^a^	1.83 ± 0.25 ^a^*^+^^‡^
HOMA-IR	5.21 ± 0.48 ^c^	7.06 ± 0.85 ^b^	6.95 ± 1.04 ^b†^	5.84 ± 0.88 ^c^	8.66 ± 1.44 ^a^	9.01 ± 1.30 ^a^*^+^^‡^
Total cholesterol (mg/dL)	195 ± 8.37 ^c^	224 ± 10.9 ^a^	206 ± 6.95 ^b^	162 ± 9.83 ^d^	189 ± 10.6 ^c^	193 ± 10.1 ^c^*^+^^‡^
HDL (mg/dL)	52.5 ± 2.98 ^a^	51.3 ± 2.35 ^a^	53.6 ± 5.05 ^a^	40.7 ± 5.34 ^b^	43.7 ± 4.10 ^b^	41.7 ± 5.12 ^b^*^+^
LDL (mg/dL)	115 ± 7.35 ^c^	150 ± 8.68 ^a^	110 ± 6.51 ^c†^	95.4 ± 5.47 ^d^	110 ± 8.47 ^c^	128 ± 10.5 ^b^*^+^^‡^
Triglyceride (mg/dL)	138 ± 11.8 ^b^	118 ± 9.75 ^d^	111 ± 8.81 ^d†^	135 ± 11.9 ^b^	176 ± 15.9 ^a^	128 ± 9.47 ^c^*^+‡^

Mice were randomly assigned to the following six groups: (1) Sham operation of cholecystectomy (Sham) + low fat/high carbohydrate diet (LFD), (2) Sham + high fat diets (HFD), (3) Sham + HFD + 2% cholestyramine (BAS), (4) cholecystectomy (GBX) + LFD, (5) GBX + HFD, and (6) GBX + HFD + BAS. Values represented means ± standard deviations (*n* = 10). * Significant GBX effect by a two-way ANOVA at *p* < 0.05. ^+^ Significant treatment effect by a two-way ANOVA at *p* < 0.05. ^†^ Significant treatment effect in Sham rat by a one-way ANOVA at *p* < 0.05. ^‡^ Significant treatment effect in GBX rat by a one-way ANOVA at *p* < 0.05. ^a,b,c^^,d^ Different letters in each variable indicate significant differences in all groups at *p* < 0.05.

**Table 3:** Glucose and lipid metabolism and inflammation in the liver.

	Sham + LFD (*n* = 10)	Sham + HFD (*n* = 10)	Sham + HFD + BAS (*n* = 10)	GBX + LFD (*n* = 10)	GBX + HFD (*n* = 10)	GBX + HFD + BAS (*n* = 10)
Hepatic glycogen (mg/g tissue)	88.8 ± 10.4 ^a^	69.0 ± 6.92 ^b^	71.3 ± 10.4 ^b^^†^	69.3 ± 4.81 ^b^	61.2 ± 6.69 ^c^	59.5 ± 6.92 ^c^*^+^^‡^
Hepatic triglyceride (mg/g tissue)	195 ± 14.1 ^c^	205 ± 17.3 ^c^	265 ± 18.3 ^a†^	186 ± 18.9 ^c^	226 ± 20.2 ^b^	245 ± 21.7 ^ab^*^+^^‡^
Hepatic *TNF-α* mRNA (AU)	0.88 ± 0.11 ^d^	1 ± 0 ^c^	0.97 ± 1.06 ^cd†^	1.05 ± 0.17 ^c^	1.24 ± 0.19 ^b^	1.49 ± 0.22 ^a^*^+^^‡^
Hepatic *SREBP1c* mRNA (AU)	1.04 ± 0.16 ^c^	1 ± 0 ^c^	1.34 ± 0.22 ^b†^	1.27 ± 0.17 ^b^	1.37 ± 0.19 ^b^	1.78 ± 0.27 ^a^*^+^^‡^
Hepatic *PPAR-γ* mRNA (AU)	0.67 ± 0.16 ^d^	1 ± 0 ^c^	1.03 ± 0.15 ^c†^	0.74 ± 0.26 ^d^	1.43 ± 0.32 ^b^	1.89 ± 0.33 ^a^*^+^^‡^
Hepatic lipid peroxides (nmol/g tissue)	21.5 ± 1.97 ^d^	28.6 ± 2.35 ^c^	29.1 ± 3.07 ^c†^	33.8 ± 3.42 ^b^	38.9 ± 4.05 ^a^	39.6 ± 3.87 ^a^*^+^^‡^
Serum ALT (U/L)	29.8 ± 3.14 ^c^	29.7 ± 3.23 ^c^	28.5 ± 2.94 ^c^	34.2 ± 3.18 ^b^	39.4 ± 3.51 ^a^	38.8 ± 3.12 ^a^*^+^^‡^
Serum AST (U/L)	34.2 ± 3.03 ^a^	31.4 ± 0.92 ^b^	30.5 ± 3.04 ^b^	33.2 ± 3.23 ^ab^	32.9 ± 1.89 ^ab^	33.1 ± 3.20 ^ab^*

Mice were randomly assigned to the following six groups: (1) Sham operation of cholecystectomy (Sham) + low fat/high carbohydrate diets (LFD), (2) Sham + high fat diets (HFD), (3) Sham + HFD + 2% cholestyramine (BAS), (4) cholecystectomy (GBX) + LFD, (5) GBX + HFD, and (6) GBX + HFD + BAS. Values represented means ± standard deviations (*n* = 10). AU, arbitrary unit. * Significant GBX effect in a two-way ANOVA at *p* < 0.05. ^+^ Significant treatment effect in a two-way ANOVA at *p* < 0.05. ^†^ Significant treatment effect in Sham rat by a one-way ANOVA at *p* < 0.05. ^‡^ Significant treatment effect in GBX rat by a one-way ANOVA at *p* < 0.05. ^a,b,c^^,d^ Different letters in each variable indicate significant differences in all groups at *p* < 0.05.

**Table 4:** Relative abundance of gut microbiomes at the genus level.

	Sham + LFD (*n* = 10)	Sham + HFD (*n* = 10)	Sham + HFD + BAS (*n* = 10)	GBX + HCD (*n* = 10)	GBX + HFD (*n* = 10)	GBX + HFD + BAS (*n* = 10)
*Akkermansia*	21.02 ± 2.59 ^a^	14.68 ± 2.12 ^ab^	18.16 ± 4.29 ^a†^	19.52 ± 3.26 ^a^	10.85 ± 3.03 ^b^	8.99 ± 1.91 ^b^*^+‡^
Lachnospiraceae_unclassified	24.58 ± 2.87 ^a^	20.69 ± 2.93 ^ab^	21.00 ± 1.66 ^ab^	17.34 ± 1.48 ^b^	17.9 ± 2.13 ^b^	17.46 ± 1.88 ^b^*
*Bacteroides*	6.18 ± 1.64 ^c^	9.50 ± 1.58 ^b^	12.73 ± 2.15 ^ab†^	12.55 ± 2.90 ^ab^	11.4 ± 3.14 ^ab^	15.34 ± 2.30 ^a^*^+‡^
*Lactobacillus*	1.48 ± 0.57 ^b^	0.58 ± 0.13 ^bc^	0.12 ± 0.06 ^c†^	10.29 ± 2.26 ^a^	7.47 ± 3.56 ^a^	1.04 ± 0.60 ^b^*^+‡^
*Oscillospira*	7.87 ± 1.22 ^b^	7.58 ± 0.62 ^b^	11.25 ± 0.68 ^a†^	8.14 ± 2.69 ^ab^	6.89 ± 1.37 ^b^	7.77 ± 0.83 ^b+^
*Desulfovibrio*	9.16 ± 1.75	9.03 ± 1.26	6.50 ± 1.07	7.72 ± 1.21	9.29 ± 1.34	7.07 ± 0.70
*Bifidobacterium*	7.25 ± 4.03 ^a^	2.50 ± 0.93 ^b^	1.80 ± 0.32 ^b†^	3.75 ± 2.06 ^b^	6.57 ± 2.63 ^a^	5.15 ± 2.88 ^ab^*^+‡^
*Parabacteroides*	6.74 ± 4.01 ^a^	2.69 ± 0.40 ^b^	6.96 ± 3.48 ^a†^	2.53 ± 0.33 ^b^	2.68 ± 0.85 ^b^	2.28 ± 0.44 ^b^*
*Ruminococcus*	1.36 ± 0.20	0.85 ± 0.13	0.96 ± 0.07	1.55 ± 0.49	1.71 ± 0.36	1.034 ± 0.17
*Clostridium*	1.88 ± 0.32 ^b^	2.41 ± 0.25 ^ab^	3.42 ± 0.59 ^a†^	1.13 ± 0.09 ^b^	1.78 ± 0.43 ^b^	4.26 ± 0.71 ^a+^
*Anaerotruncus*	2.86 ± 0.76 ^a^	1.26 ± 0.26 ^ab^	0.88 ± 0.19 ^b†^	1.04 ± 0.20 ^b^	0.96 ± 0.21 ^b^	1.32 ± 0.26 ^ab^*
*Alistipes*	0.04 ± 0.01 ^b^	0.07 ± 0.02 ^b^	0.11 ± 0.03 ^ab^	0.13 ± 0.02 ^b^	0.07 ± 0.02 ^b^	0.26 ± 0.07 ^a^*^+‡^
*Dorea*	0.06 ± 0.03 ^b^	0.12 ± 0.03 ^ab^	0.05 ± 0.01 ^b^	0.11 ± 0.03 ^ab^	0.05 ± 0.01 ^b^	0.50 ± 0.13 ^a^*^‡^
*Blautia*	0.001 ± 0.001 ^b^	0.06 ± 0.03 ^a^	0.04 ± 0.03 ^a†^	0 ± 0 ^b^	0 ± 0 ^b^	0.43 ± 0.11 ^a+‡^

Mice were randomly assigned to the following six groups: (1) Sham operation of cholecystectomy (Sham) + low fat/high carbohydrate diets (LFD), (2) Sham + high fat diets (HFD), (3) Sham + HFD + 2% cholestyramine (BAS), (4) cholecystectomy (GBX) + LFD, (5) GBX + HFD, and (6) GBX + HFD + BAS. Feces were collected from the cecum after the 8-week treatment, and fecal bacterial composition was analyzed using the NGS method. Dots or bars and error bars represent the means ± standard deviations (*n* = 10). * Significant GBX effect by a two-way ANOVA at *p* < 0.05. ^+^ Significant treatment effect by a two-way ANOVA at *p* < 0.05. ^†^ Significant treatment effect in Sham rat by a one-way ANOVA at *p* < 0.05. ^‡^ Significant treatment effect in GBX rat by a one-way ANOVA at *p* < 0.05. ^a,b,c^ Different letters in each variable indicate significant differences in all groups at *p* < 0.05.

## Data Availability

The data presented in this study are available upon request from the corresponding author.

## Abbreviations

BAS: bile acid sequestrants; GBX: gallbladder removal; SREBP1c: sterol regulatory element-binding transcription factor 1c; HOMA-IR: homeostatic model assessment for insulin resistance; PPAR-r: peroxisome proliferator-activated receptor-gamma; TNF-α: tumor necrosis factor-α; SCFA: short-chain fatty acids; FXR: Farnesoid X receptor; HFD: high-fat diets; LFD: high carbohydrate diets; LDA: linear discriminant analysis; En%: energy percent; KEGG: Kyoto Encyclopedia of Genes and Genomes; KO: KEGG Orthologues; ANOVA: analysis of variance; SDs: standard deviations; LDL: low-density lipoprotein; PCR: polymerase chain reaction; H-E: hematoxylin-eosin; AB-PAS: Alcian blue-perchloric acid; NGS: next-generation sequencing.
